# Detecting change processes during an ACT-based course for enhancing university students’ well-being and study skills

**DOI:** 10.3389/fpsyg.2025.1697887

**Published:** 2025-12-19

**Authors:** Nina Katajavuori, Sara Rönkkönen, Liisa Postareff, Anni Rytkönen, Henna Asikainen

**Affiliations:** 1 Centre for University Teaching and Learning (HYPE), Faculty of Educational Sciences, University of Helsinki, Helsinki, Finland; 2 Unit for Research and Development of Higher Education Pedagogy, HAMK Häme University of Applied Sciences, Hämeenlinna, Finland; 3 Department of Computer Science, School of Science, Aalto University, Espoo, Finland

**Keywords:** university students, well-being, transtheoretical model, time and effort management skills, psychological flexibility, study-related burnout

## Abstract

**Introduction:**

Acceptance and Commitment Therapy (ACT)-based interventions have proven effective in supporting student well-being. However, the change processes occurring during such interventions remain unclear. This study explored changes reported by university students participating in an ACT-based online course which also included study skills.

**Method:**

The data included reflection journals and questionnaire responses on psychological flexibility, time and effort management, and study-related burnout at the start and end of the course.

**Results:**

Both qualitative and quantitative findings indicated numerous positive effects. Utilizing the transtheoretical model, we identified four change profiles among the students: No Change, Change in Thinking, Initial Change and Change in Behavior profiles. These profiles reflected varying levels of behavioral changes related to well-being and studying supported by differing processes of psychological flexibility. While profiles did not differ significantly at the beginning of the course, there was a significant overall increase in psychological flexibility and time and effort management skills, alongside a decrease in study-related burnout dimensions—except for Inadequacy, which increased in the Initial Change profile.

**Discussion:**

Students in Initial Change profile may benefit from additional support to enhance their well-being and study practices. These findings provide insights into the diverse change processes among students, highlighting the need for tailored support to encourage behavioral change. Future research should examine individual differences to better target interventions for students at different stages of change.

## 1 Introduction

University students can experience several academic and personal challenges during their studies (Friedlander et al., 2007; Pedrelli et al., 2015; Yu and Wright, 2016; Mofatteh, 2020). One internationally growing concern has been the decline in university students’ well-being, as well as increasing mental health problems (Evans et al., 2018; Auerbach et al., 2018). Worryingly, this is backed up by evidence suggesting that student mental health continues to deteriorate over time (Emmerton et al., 2024). These issues hinder students’ academic progress, success, and weaken their performance during their studies (McGillivray and Pidgeon, 2015; Asikainen et al., 2022; Emerson et al., 2023; Baalmann, 2023), potentially leading to increased stress and mental health challenges related to their studies (Mofatteh, 2020).

A central factor threatening students’ well-being is study-related burnout (Salmela-Aro et al., 2009). Various elements affect it, but currently one promising factor for decreasing burnout symptoms and promoting well-being is psychological flexibility (Zhang et al., 2018). The origin of psychological flexibility is in Acceptance and Commitment Therapy (ACT), and it describes people’s ability to take actions which are in line with their values, despite the negative thoughts and feelings that may occur (Chawla and Ostafin, 2007; Hayes et al., 2006). Decades of research have demonstrated that ACT-interventions which target on developing psychological flexibility have a profound impact in improving well-being and life satisfaction, as well as decreasing a wide range of psychological problems such as depression, anxiety and stress for different populations including higher education students (for review studies and meta-analyses see Bai et al., 2020; Gloster et al., 2020; Howell and Passmore, 2019; Towey-Swift et al., 2023). Promising results have additionally been observed from ACT-based intervention studies that incorporate study skill training (Asikainen and Katajavuori, 2022; Katajavuori et al., 2023). In higher education contexts, it has been shown that study skills play a significant role in student well-being. Time and effort management skills have been shown to play a crucial role in promoting students’ well-being, academic success and steady progress (Hailikari and Parpala, 2014; Asikainen et al., 2020; Hailikari et al., 2018; Asikainen and Katajavuori, 2022).

However, there is increasing evidence that group averages do not describe individuals in terms of determinants of mental health and well-being (Ciarrochi et al., 2024). In other words, we know that interventions do not affect all students in the same way. Thus, we need more person-oriented research on the effects of ACT-based interventions to better understand the individual processes of change. One widely recognized theoretical framework to capture the change in one’s behavior is the transtheoretical model (TTM), developed by Prochaska and DiClemente (1982). The model describes the processes of behavior change, and the steps involved in moving towards the desired outcome. The model has been widely used in health promotion interventions, but no studies have used it in studying the behavior changes in ACT-based interventions. Psychological flexibility and its subprocesses are seen as a mechanism for behavior change (Ong et al., 2024) and thus we expect the TTM model to be suitable for analyzing the change processes in the course. We expect psychological flexibility processes to foster the change process.

This mixed methods study aims first, to explore students described behavior changes regarding their well-being and studying during an ACT-based well-being and study skills course using transtheoretical model as a framework. In addition, the aim is to examine how these change profiles differ in their initial scores as well as changes in their scores on psychological flexibility, time and effort management skills and study-related burnout.

### 1.1 Study-related burnout

Study-related burnout can be defined through three dimensions, namely exhaustion, cynicism, and inadequacy (Salmela-Aro et al., 2009, 2022; Salmela-Aro and Kunttu, 2010). Study-related exhaustion refers to feelings of being burdened resulting from overtaxing study demands (Salmela-Aro and Read, 2017). Cynicism, in turn, refers to lack of interest and decreased feelings of interest, and meaningfulness in studying (Salmela-Aro and Read, 2017; Schaufeli et al., 2002). Feelings of inadequacy and a lack of study-related efficacy refer to feelings of incompetence and poor achievement in one’s studies (Maslach et al., 2001; Schaufeli et al., 2002; Salmela-Aro et al., 2009). All aspects of study-related burnout have been found to negatively affect students’ dedication to university studies (Salmela-Aro and Upadyaya, 2017), are related to depressive symptoms and lower levels of well-being (Salmela-Aro et al., 2009), and reduced academic achievement (Asikainen et al., 2022; Madigan and Curran, 2021; Salmela-Aro and Upadyaya, 2017).

### 1.2 Time and effort management

One aspect affecting student well-being and study-related burnout is time and effort management. Time and effort management skills include a student’s capability to set goals, study according to those goals, prioritize tasks, and manage their time effectively (Entwistle et al., 2001). These skills are crucial in promoting students’ progression in their studies (Rytkönen et al., 2012; Asikainen et al., 2020; Häfner et al., 2015). However, many students find developing their time management skills during their studies very challenging (Parpala et al., 2017), which can then lead to further problems like procrastination behavior and a lack of planning for academic activities (Asikainen et al., 2013; Fentaw et al., 2022). Students who struggle with time and effort management often experience high levels of stress, exhaustion, and a general lack of interest in their studies (Heikkilä et al., 2012). In addition, research shows that students who experience troubles in time and effort management skills score higher on experiences of study-related burnout compared to those with good study skills (Asikainen et al., 2020; Asikainen and Katajavuori, 2023; Parpala et al., 2021). Time management skills can be developed through simple practices such as planning and setting goals underscoring the importance of teaching these abilities to students (Kitsantas et al., 2008). Notably, some successful targeted interventions have already been developed to improve students’ study, and time and effort management skills in universities (e.g., Häfner et al., 2015; Biwer et al., 2020).

### 1.3 Psychological flexibility

The concept of psychological flexibility, rooted in Acceptance and commitment therapy, can be defined as people’s ability to take actions which are in line with their values despite of the negative thoughts and feelings one might have (Chawla and Ostafin, 2007; Hayes et al., 2006). Thus, people with high psychological flexibility can act according to what really matters to them, are willing to confront and accept their feelings and emotions with a non-judgement attitude, and to take an observer perspective to them (Bond et al., 2010).

Psychological flexibility consists of six interconnected processes, each capturing skills that can be practiced and developed. One of the central processes in psychological flexibility is defusion, which indicates a person’s ability to look at their own thoughts as separate parts of themselves, and the ability to see thoughts as just thoughts as opposed to truths or factual representations of the world or them (Hayes et al., 2006). Acceptance means that all fluctuations of feelings, emotions and experiences and welcomed as they arise without the aim to deny them (Biglan et al., 2008; Blackledge and Barnes-Holmes, 2009) and contact with the present moment is a perquisite for embracing and recognizing all experiences. Seeing self as a context refers to a person’s ability to explore inner experiences more objectively (Hayes et al., 2006). Committed actions refers to one’s ability to take value-based action, with accepting those distressing thoughts and emotions which may occur and try to hinder this action and still act according to what really matters to them (Blackledge and Barnes-Holmes, 2009). Value-based action and behavior are necessary for life satisfaction and for the experience of a meaningful existence (Hayes, 2019), which in turn increases one’s well-being. The sub-processes of psychological flexibility trained during an ACT-based intervention can foster health-promoting behavior (Moffitt and Mohr, 2015; Bricker et al., 2017). For example, acceptance of feelings and thoughts, awareness of decision-making thoughts, and commitment to chosen values have been recognized as essential components in fostering behavior change (Forman and Butryn, 2015; Räihä et al., 2024). Thus, further applications of fostering psychological flexibility have been called for to promote behavior change and well-being (Zhang et al., 2018). Thus, psychological flexibility could be seen as a key factor in fostering behavior change as it helps individuals to respond to situations in ways that help to reach the valued goal and the skills in psychological flexibility are particularly useful when challenges arise during goal pursuit (Doorley et al., 2020).

To conclude, psychological flexibility can be viewed as a fundamental to well-being and health and includes a wide range of abilities to recognize and adapt to different demands which arise, maintain balance among important life domains, and further, be open and committed to action that are in line with one’s values (Kashdan and Rottenberg, 2010). Interventions aiming to increase psychological flexibility have a great potential to help people in finding greater efficacy and fulfilment in their daily lives (Kashdan and Rottenberg, 2010).

### 1.4 Transtheoretical model

One way to explore the process of change in behavior related to well-being and time and effort management skills is through the widely recognized framework of the Transtheoretical Model (TTM) (Prochaska and DiClemente, 1982). It describes one’s behavior change towards the desired goal; it is used especially in the context of enhancing one’s health-promoting behavior. The model has previously been applied to several health behaviors like smoking cessation, physical exercise, dieting, and chronic diseases (Prochaska et al., 2008; Hashemzadeh et al., 2019). The model has also been applied in educational contexts to improve study techniques or to decrease procrastination during studies (Grant and Franklin, 2007; Grunschel and Schopenhauer, 2015). However, to the best of our knowledge model has not yet been used to analyze changes in the ACT context, although it has been suggested that the transtheoretical model can serve as a valuable psycho-educational tool, which could be used to educate individuals on improving their study habits and facilitating the change process, while also helping to identify different student profiles that represent various stages of the process (Grant and Franklin, 2007).

TTM suggests that behavior change is a process involving various stages, with individuals at different stages of change and readiness (Prochaska and DiClemente, 1982). The model further assesses individuals’ readiness for a new healthier behavior and highlights that change is a process, not a singular event (Boonroungrut and Fei, 2018). According to the model, change occurs in five different stages (Prochaska and DiClemente, 1982; DiClemente et al., 1991). The first stage is called *precontemplation*, meaning that one is not ready for a change, unaware of the need for a change or not necessarily even considering the change. The second stage is *contemplation*, meaning that one is preparing themself for a change, by becoming aware that there is a need for change and begin to question their current behavior (Boonroungrut and Fei, 2018). The third phase is called *preparation*, which means that one is ready to take actions towards the change in one’s behavior. One has decided to make a change and may start to take small steps toward the change (Prochaska and DiClemente, 1982). After this stage is phase called *action,* which involves active engagement to the new, better behavior. In this stage, one has made efforts to change their behavior and has also achieved success. Once the change has been made, the *maintenance* phase focuses on securing the progress which has been achieved and further avoiding relapses. This stage includes sustaining the new behavior over the long term (Prochaska and DiClemente, 1982). These stages in the process are not always linear. It is likely that people can move between the stages, and it is possible to return to a previous level as well. This is especially the case if they face challenges or relapses (Adams and White, 2005).

According to the TTM there are three key factors controlling the transfer between different stages for change which are process of change (PC), decisional balance (DB) and self-efficacy (SE) (Prochaska et al., 1994; Herrick et al., 1997; Prochaska et al., 2008). These factors can be used to differentiate between the individuals in the stages of the change and to display their progress during the process and stages (Prochaska et al., 2008). Process of change refers to cognitive, emotional and behavioral strategies that facilitate movement from one stage to another. Five central processes of change were identified by Prochaska and DiClemente (1982): (1) Consciousness raising increases awareness of the behavior and its consequences, while (2) dramatic relief involves emotional engagement that supports motivation. (3) Self-liberation reflects strengthening commitment and belief in one’s ability to change (4) Counterconditioning and stimulus control involve replacing maladaptive responses and modifying environmental cues, whereas (5) contingency management changes the reinforcement patterns that maintain behavior. Psychological flexibility processes can be related to these processes. Present moment awareness is very close to consciousness raising as they both emphasize awareness of behavior and thoughts. The catharsis process involves accepting, recognition, and opening up to difficult emotions and feelings in change which are in line with defusion and acceptance. The self-liberations phase involves committing or recognizing values, which is a central process in psychological flexibility. Counter conditioning and stimulus control as well as contingency management can involve recognizing one’s values and taking value-based actions towards the wanted behavior.

Decisional balance refers to the balance between drawbacks and benefits where individual is weighing of the pros and cons of changing (Prochaska et al., 1994; Herrick et al., 1997; Prochaska et al., 2008). Psychological flexibility skills on focusing on the present moment in difficult situations and making choices on one’s actions away from autopilot and more towrads one’s values. Furthermore, self-efficacy refers to one’s confidence regarding their capability to do the actions needed to change behavior towards more favourable behavior and it usually increases across the stages of change (Grant and Franklin, 2007; Keller et al., 1999). It was shown that self-efficacy has been shown to be the strongest predictor of maintaining health behavior for a longer period (Prochaska and DiClemente, 1982). In addition, psychological flexibility has been shown to predict self-efficacy in higher education students (Jeffords et al., 2020).

As TTM describes the stages and processes through which individuals change their behavior, it offers a useful framework for exploring the individual change process among university students in an ACT-based course. Psychological flexibility supports behavior change by helping individuals to stay present, open up to difficult internal experiences, and engaging in value-based actions even in the presence of contradictory thoughts or emotions (Hayes and Pierson, 1999). Individuals can improve their behavior change through committed actions which are based on their values, while accepting the contradicting thoughts which may occur. For that reason, it has been suggested that behavior change interventions could utilize ACT and enhance one’s psychological flexibility skills in fostering change (Zhang et al., 2018).

## 2 Aims of the study

The aim of this study is to explore what kind of behavior changes happen during an ACT-based intervention to improve well-being and studying. To reach the aim, the following research questions were formulated:

- What kind of change profiles can be identified among the students regarding their perceived benefits of the intervention for their well-being and studying?
- How do psychological flexibility, time and effort management skills and study-related burnout change during the intervention and how do the change profiles differ in their initial scores as well as changes in their scores on them?

## 3 Methods

### 3.1 Context

The aim of the seven-week online course Towards better well-being and studying (3 ECTS) was to enhance participants’ psychological flexibility and through this, enhancing their well-being. Further, the course aimed to enhance students’ skills in identifying factors related to well-being and studying, as well as their ability to utilize various tools to support these. The third aim of the course was to foster students’ study skills through time management training. The course content applied Acceptance and Commitment Therapy (ACT) methods, meaning that the basis of the course was to practice the skills in psychological flexibility. Time and effort management training was combined to the course, to enhance students’ well-being, learning and studying (*authors*).

The course consisted of a pre-assignment week and six course theme weeks. Each course week focused on a theme related to psychological flexibility or study skills. The six course themes were Values, Relaxation and concentration, Power of thoughts, Sleep, exercise and study techniques, Self-compassion, and Living by your values *(authors)*. The course material included recorded video introductions to the theme, reading material, exercises as well as individual and group reflective assignments. Individual assignments included for example exercises where students practiced concentration, being present and defusion with for example Leaves on the Stream –metaphor and Passengers on the Bus (Morris and Oliver, 2013). Time management was trained through a structured process. Participants tracked their time use for 1 week during the pre-task week, reflected on this during the subsequent values week and then set goals for their time usage based on their personal objectives which were defined in the beginning of the course *(authors)*. Each week included a peer group meeting with instructions and guiding questions *(authors).* Peer groups were allowed to choose themselves whether to meet online or face-to-face.

At the end of the course, the final individual assignment was to write a report with reflections on the course, as well as its meaning for the students themselves and their studies. The students were instructed to return a reflective report, length 2–3 pages. They were instructed to reflect on how the intervention had affected their well-being and studying and if not why, and what concrete actions they planned to take to improve their studies and well-being. The report was a compulsory part for passing the course. Additionally, students were asked to evaluate their well-being at the beginning of the course and again at the end of the course by answering Likert scale statements related to their well-being, psychological flexibility, study skills, and burnout risk. They were asked to reflect these evaluations in their learning report.

### 3.2 Data collection

The study was conducted among Higher Education students who took part in the Towards Better Well-being and Studying course in the Spring 2022 in Finland. All the participants of the study were adult, being Bachelor’s or Master’s students in various disciplines including arts, business, technical, social and life sciences. The participants came from two research-intensive university and one university of applied sciences. Their age or gender were not asked for systematically. Altogether 103 students gave their consent to participate in the research and returned their written learning report at the end of the course.

The study adopted a mixed-methods approach using exploratory sequential mixed-method design, consisting of an initial qualitative phase followed by a subsequent quantitative phase (Creswell and Plano Clark, 2018). In this study, mixed methods approach was applied to make a complete analysis of the change processes by examining personal qualitative experiences as well as quantitative outcomes during the course (Creswell and Inoue, 2025). The qualitative data were collected by using the reflective reports that the students wrote at the end of the course. The data were used to qualitatively identify students’ change profiles regarding their perceived benefits for their well-being and studying. The quantitative data were collected with Likert scale surveys at the beginning of the course, meaning the pre-task week, and at the end of the course, meaning during the last week of the course (seventh week). Quantitative data were used to examine how psychological flexibility changed over the course and to explore how the change profiles identified in the qualitative analysis differed in terms of psychological flexibility, study-related burnout, and time and effort management skills. Out of the 103 participants who returned their written learning report at the end of the course, 7 students did not answer either the first or the second survey. Thus, the quantitative data consisted of 96 students.

Psychological flexibility was measured by using Comprehensive Assessment of Acceptance and Commitment Therapy Processes (CompACT) questionnaire (Francis et al., 2016) and organized studying measuring time and effort management skills was measured by four items used in the HowUlearn questionnaire (Parpala and Lindblom-Ylänne, 2012) with a 5-point Likert scale. Study-related burnout was measured with the nine-item Study Burnout Inventory (SBI-9), and it consisted of all three dimensions of burnout, namely exhaustion, cynicism and inadequacy (Salmela-Aro and Read, 2017) with a 6-point Likert scale. The measures which were used in this study have shown good internal consistency and validity in earlier research (Parpala and Lindblom-Ylänne, 2012; Francis et al., 2016).

According to local legislation, this study did not require prior ethical review. None of the conditions necessitating an ethics review in Finland, as defined by the Finnish National Board on Research Integrity (TENK, 2019), were met in this study. The course participants were informed about the research and its purpose at the beginning of the course. Students were asked for consent to use the data they produced during the course for research purposes, and they had the possibility to change their mind during the course. Participation in the research was voluntary and did not affect any way to completion of the course. All study participants also gave consent to use their learning reports in the study which were used for the qualitative part of this study to explore what kind of experiences and changes the students described in their reports. All data were pseudonymized before analysis. All names and student numbers were removed from the report files’ contents and metadata. Responses were coded with running numbers so that individual students could not be identified and still, the respondents could be separated from each other.

### 3.3 Analysis

The entire qualitative research process was guided by the Standards for Reporting Qualitative Research (SRQR) (O’Brien et al., 2014) and the Critical Appraisal Skills Programme (CASP) criteria for qualitative research (Long et al., 2020), ensuring methodological rigor and transparency. According to the mixed-method design used in this study, the aim was first to examine students’ personal qualitative experiences. First, all the reports were qualitatively analyzed with abductive content analysis using the transtheoretical model. A person-oriented approach (Bergman and Andersson, 2010; Mäkikangas and Kinnunen, 2016; Woo et al., 2018) was utilized to detect qualitative change categories regarding well-being and/or studying. The process proceeded as follows: In the first step of the qualitative analysis, the first, second, and fourth authors familiarized themselves with the data by carefully reviewing the reports multiple times. After gaining a holistic view of the content, the authors identified the data segments relevant to the research question, that is, the segments in which the participants described the benefits of the intervention for their well-being and studying. After that, the transtheoretical model (Prochaska and DiClemente, 1982) was utilised as a guiding framework for an iterative categorization process to detect distinct change categories in students’ experiences of psychological flexibility, well-being, and time management. The first step of the analysis resulted with three main change categories: *No changes or insights*, representing a (pre)contemplation stage; *Change in thinking,* representing a contemplation stage, and *Initial* c*hange*, representing both, preparation and *Change in behavior*.

In the second step of the analysis, the first and second authors re-distributed the texts for a second reading and discussed selection criteria for the categories, agreeing on the use of subcategories. Reliability was ensured through ongoing discussions about codes with multiple interpretations. The biggest category, which included behavior change, consisted of students who reported making concrete efforts to change their behavior, with varying degrees of success, from minor changes including single, small not long lasted single change, such as implementing some time management technique (preparation stage) up to making multiple and extensive changes systematically for a longer time during the intervention (action stage). Two subcategories emerged within the Behavior Changes category: *Initial Change* and *Changes in Behavior*. Final coding was reached through mutual agreement and further validated through discussions with all authors. The final counts for each category were: No Changes or Insights (4), Change in Thinking (30), Initial Change (43), and Change in Behavior (26). After assigning students to the main change categories, the first and second author reanalyzed the learning reports within each category, coding all the mentions related to students’ insights and behavior changes. This analysis yielded seven subcategories related to behavior changes and eight related to insights. Finally, the first author reanalyzed a portion of the learning reports to verify the analysis, eliminating any ambiguities through thorough discussions on classification criteria. The categories were quantified at the end of the analysis process.

Survey data from the beginning and end surveys were combined to review students’ perceived changes related to well-being and studying during the course. After the first qualitative analysis phase of the reports, the identified categories of change were included in the survey data and SPSS was used for the second research phase quantitative analysis. Normality of the scales measuring study-related burnout and its components, psychological flexibility and time management was explored based on skewness and kurtosis and morality curves which showed that the data was normally distributed to use parametric tests. The reliability analysis for the scales was made with Cronbach’s alpha since the data were too small for confirmatory factor analysis, and these scales have been widely used, and their validity has been shown in various studies. The relationship between psychological flexibility, study-related burnout, and organized studying was analyzed with Pearson’s correlations. Differences between the profiles in their initial values, as well as changes in burnout, organized studying, and psychological flexibility, were analyzed using repeated measures ANOVA. The analysis considered time, group, and time*group interactions, with Bonferroni *post hoc* tests for further comparisons.

## 4 Results

### 4.1 The change profiles identified among the students and the description of the profiles (R1 and R2)

The results showed that there was a variation in the behavior changes related to students’ psychological flexibility, well-being and time management in their descriptions in the end of the course. We found four change profiles which describe the changes that occurred during the course (see Table 1), namely *No changes or insights; Change in thinking; Initial change and Change in behavior.*

**Table 1:** The change profiles detected.

Change profile	*N*	%	Characteristics of the profile
No changes or insights	4	4	*Precontemplation* stage; being unaware of the need for a change, not having the necessary resources (e.g., time) to make changes in one’s behavior
Change in thinking	30	29	*Contemplation* stage; making insights and realisations and becoming aware of a need for change, preparing and getting ready for it
Initial change	43	42	*Preparation* stage; being ready for taking actions towards the change, having decided to change one’s behavior, having possibly started to take small steps toward the change
Change in behavior	26	25	*Action* stage; starting to make concrete efforts to change one’s behavior, having a plan on how to sustain the new behavior

#### 4.1.1 No changes or insights

The first profile was *No changes or insights* profile, which represents a *precontemplation* stage, consisted only four students who did not report changes during the course. Typical for the experiences of these students was that they felt that the timing of the course was not good for them and reported of being too stressed or busy during the course to invest to the course and its aim to foster studying and well-being like one of these students wrote:

*This course, so to speak, went by the wayside - I always ended up doing the assignments at the last minute. Therefore, I can honestly say that I should have taken the course at a different time. (A 7)*

#### 4.1.2 Change in thinking

The second profile was *Change in thinking* profile, representing a *contemplation stage*. These students (*n* = 30) had awakened to new understandings; they reported that they had learnt new things from themselves and had learnt to understand different themes related to their well-being and studying. Most often, the students in this profile (*n* = 21, 70%) mentioned that they had realized the importance of psychological flexibility and its sub-processes and further, its importance in fostering their well-being and studying. These students had understood the importance of breaks, breathing and concentration for well-being, had understood the significance of seeing thoughts as thoughts and had been thinking about what really mattered to them in their life.

12 of the students in this profile (40%) reported that they had realized they needed to improve their skills in time management and studying. They had learnt to understand their patterns related to studying and had realized the importance of good time-management skills:

*“At the top of my mind is the effect of procrastination in life, time management, and experiencing happiness and stress-freeness. I realized that my typical procrastination prevents me from achieving stress-free and happy life.” (A25)*

Furthermore, 14 students (47%) in this profile described that they had understood the significance of well-being and better life habits during the course and had gained new insights about themselves:

*“Among other things, I have realized that well-being is not a goal in itself, but rather a journey to be taken. Well-being is not static in its state, but you really have to work for it and make an effort – you reflect a lot on what you have learned and what you have previously learned, but do not tell about the changes.” (A65)*

For the students belonging to the *Change in thinking* profile, these realizations lead to realizing the need for change and that after these insights it would be possible to change their behavior. However, students in this profile did not report concrete changes in their behavior related to their well-being or studying. Many mentioned that translating insights into practical action is challenging and would require more time for processing what they had learnt, and that the timing of the course proved too challenging, for example, due to other academic commitments. Additionally, some students mentioned that they had expected the course to provide more practical tips for studying, so the broader focus on well-being came as a surprise to them.

#### 4.1.3 Initial change

The third profile was *Initial change* profile, representing *preparation stage.* These students (*n* = 43) reported small steps and single changes in their behavior. All these students described the insights and learning that occurred during the course. A change in thinking happened among these students and preceded or occurred simultaneously with concrete changes in behavior. Most often students (*n* = 26, 60%) reported in their reports that they had realized new insights about psychological flexibility and its subprocesses – for example they had realized the power of thoughts and had realized how hard they were for themselves. Students had also realized the significance of time management and study skills (*n* = 22, 51%) and recognized the need for improvement and took some actions in this area. 11 (26%) students had also reported that during the course they had understood the significance of taking care of one’s well-being; like the importance of sleeping and exercising.

The students in this profile had also made single changes in their actions concerning either their studies, their personal well-being, or thinking, or all of them. Changes had been initiated during the course related most often to studying (*n* = 29, 67%) including time management and they had practiced goal setting in their studies:

*“I did not fully reach the course goal, but some progress may have been made. At the beginning of the course, I noticed that my screen time was high, and I have tried to draw attention to it. Also, I’ve been working more and I have occasionally used different study techniques, such as the Pomodoro technique, to improve my concentration.” (A70)*

26 students (60%) reported that they started to practice their skills in psychological flexibility. For example, they had started to implement presence and concentration exercises in their everyday life, practiced managing negative thoughts, and implemented self-compassion. Several students reported that they had started to think differently about their own performance expectations; for example, by lowering the bar in their studies and realizing that less is enough. Actions aligned with their values had also begun to be implemented during the course; a few students described how they had started to focus on important personal relationships in their daily life, which they noticed had supported their studies and well-being.

*“[…] As the course progressed, I noticed how many internal actions were the pillars of support for achieving such an everyday goal. Reflection on one’s own values, thought control and self-compassion would not have been the first thing that came to mind when, thought how to clarify my studies.” (B 3001_6)*

It was typical in the descriptions of students in this profile that they had noticed how slow and challenging the process of behavior change is. They had also considered the obstacles to change, and many recognized that their own ways of thinking and routines were so strong or rigid that changing them was difficult. They often felt uncertainty and noted that they were only at the beginning of their change process:

*“During the course, I noticed that although I have not yet reached my goal, I have better perceived its existence. The “magnitude” of the problem clearly surprised me. It is not only a question of my will, but I must also work towards my goal and introduce* var*ious aids. The problem of time management cannot be solved overnight, because it has not arisen overnight.” (A75)*

#### 4.1.4 Change in behavior

The fourth profile was *Change in behavior*, representing the *action stage*. Compared to the third profile, students (*n* = 26) in this profile reported several changes that they had implemented to their daily life during this intervention. The changes implemented related to their studying or well-being were either few or several changes. Characteristic of these changes was that they were reported to be permanent and implemented in their everyday life. The tone of the reports was positive and enthusiastic.

In this profile, the students reported most often (*n* = 23, 88%) how they had implemented the skills in psychological flexibility in their everyday lives. Typically, these students reported having started to practice self-compassion and a compassionate attitude towards themselves. Additionally, they described that they had started to manage their negative thoughts and emotions as well as concentration skills and being mindfully present:

*“I have noticed that I often live in the past or the future, and simple breathing exercises every day, for example, have smoothly returned me to the present moment. When breathing, I cannot be like the here and now, only the moment matters.” (A 17)*

The students in this profile reported also many changes concerning their time usage and studying (*n* = 21, 81%); they had started to do a schedule for their studies and to prioritize their time-usage:

*“Now, however, I have always put my family first and I feel that this course has had a huge impact on my decision because in the beginning, I had to think about things that were important to me, and I understood that family is the most important thing to me in my life. I’ve also seen my grandparents a lot more and called them once a week. They have been very happy and grateful for this.” (C_01)*

Over half of the students (*n* = 16, 62%) also reported that they had started to put effort in their own well-being, for example through starting to exercise more or getting more sleep:

*“Increasing physical activity is successful, I have set up a pedometer, it needs to take 8,000–10,000 steps a day and I’ve also been to the gym. I’m leading a pretty hectic life at the moment, but exercise is also worth planning to get it done. Exercise has made me feel good, and fitness has quickly done so has begun to rise. In nutrition, I have made small changes to a healthier one.” (B_3001_3)*

The students in this profile reported the change in their thinking which fostered the change in their behavior. Most often the change in thinking and the insights learnt during the course were related to self-compassion and being too hard towards themselves or they had realized how negative and rude thoughts they had about themselves. Furthermore, these students had taken advantage of the course exercise concerning the power of thoughts and self-compassion and had started to work with their thoughts in a more comprehensive way, which in turn often led to a more compassionate attitude towards oneself. The changes obtained during the course were facilitated by following their time usage and practicing self-compassion.

### 4.2 How do the changes differ between profiles?

Next, we explored how the changes differ between profiles found. Missing value analysis showed that there were no separate missing values in the data. Next, Cronbach’s alphas were calculated to the scales measuring psychological flexibility, organized studying and study-related burnout, and its three components. Cronbach’s alphas ranged generally between 0.751–0.923 (see Table 2). Only the Inadequacy scale in the second measurement had a lower but acceptable value (*α* = 0.627). The correlation analysis showed that all the measured components were related to each other (see Table 3). Time and effort management correlated positively with psychological flexibility and negatively with study-related burnout and all its components. In addition, psychological flexibility was negatively related to study-related burnout composing exhaustion, cynicism, and inadequacy.

**Table 2:** Descriptive statistics and Crohnbach’s alphas of the scales.

Measure	M1	sd	*α*	M2	sd	*α*
Time management	3.03	0.90	0.751	3.51	0.86	0.803
Psychological flexibility	3.62	0.91	0.898	4.00	0.92	0.923
Exhaustion	3.25	1.24	0.829	2.86	1.10	0.795
Cynisism	2.87	1.45	0.890	2.46	1.25	0.850
Inadequacy	3.80	1.52	0.766	3.68	1.27	0.627
Study-related burnout	3.25	1.19	0.903	2.90	1.00	0.867

**Table 3:** Correlations between items measuring psychological flexibility, time management and study-related burnout and its components in the first measurement (lower) and in the second measurement (higher).

Measure	TM	PF	SB	EX	CY	IN
TM Time and effort management	1	0.559***	−0.421***	−0.279**	−0.352***	−0.474***
PF Psychological flexibility	0.507***	1	−0.612***	−0.527***	−0.521***	−0.496***
SB Study-related burnout	−0.453***	−0.610***	1	0.859***	0.832***	0.793***
EX Exhaustion	−0.282**	−0.554***	0.875***	1	0.507***	0.549***
CY Cynisism	−0.438***	−0.518***	0.875***	0.592***	1	0.562***
IN Inadequacy	−0.505***	−0.504***	0.842***	0.607***	0.681***	1

***, *p* < 0.001; **, *p* < 0.01; *, *p* < 0.005.

**Figure 1:**
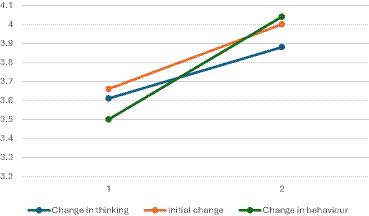
Changes in psychological flexibility across different profiles. 1 = measurement in the beginning of the course. 2 = measurement at the end of the course.

**Figure 2:**
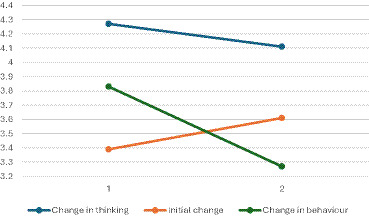
Changes in inadequacy across different profiles. 1 = measurement in the beginning of the course. 2 = measurement at the end of the course.

Next, we wanted to analyze how different profiles differed in relation to psychological flexibility, time and effort management, and study-related burnout at the beginning of the intervention. The results showed that the profiles did not statistically significantly differ from each other at the beginning of the course (*p* > 0.067). Finally, we analyzed how the changes in these dimensions differed between the profiles to explore if the level of changes would also be seen in the quantitative data. In this analysis, we excluded the *No changes* profile since it had just four students. The repeated ANOVA first showed that the effect of time was significant for all aspects measured except for Inadequacy (*p* = 0.145, see Table 4). Thus, there was a significant increase in psychological flexibility and time and effort management and a significant decrease in all dimensions of study-related burnout during the course, except for Inadequacy. The effect sizes can all be considered small. The Time x profile effects showed that the changes in psychological flexibility and inadequacy differed between the profiles (see Table 5). In all the profiles there was an increase in psychological flexibility but in the profile C*hanges in behavior* the increase was bigger than in the other profiles (see Figure 1). In addition, concerning inadequacy, the profile *Initial change* differed from the others because there was an increase in inadequacy as in the other profiles inadequacy decreased; a slight decrease in the profile *Changes in thinking* and a bigger decrease in the profile *Change in behavior* (see Figure 2).

**Table 4:** Repeated measure ANOVA.

Measure	Time			Profile			Time x profile		
	*F*	*p*	*η^2^*	*F*	*p*	*η^2^*	*F*	*p*	*η^2^*
Psychological flexibility	36.41	**<0,001**	0.290	0.190	0.827	0.004	3.86	**0.025**	0.080
Time and effort management	66.07	**<0.001**	0.426	3.21	0.067	0.941	1.44	0.241	0.031
Study-related burnout	25.97	**<0.001**	0.226	0.72	0.491	0.016	1.40	0.252	0.031
Exhaustion	23.60	**<0.001**	0.210	0.58	0.562	0.013	0.36	0.693	0.008
Cynicism	22.60	**<0.001**	0.202	2.20	0.117	0.047	0.150	0.861	0.003
Inadequacy	2.17	0.145	0.024	2.78	0.067	0.059	3.87	0.**024**	0.080

**Table 5:** Changes in psychological flexibility and inadequacy differed between the profiles.

Measure	Change in thinking (*n* = 28)		Initial changes (*N* = 38)		Change in behavior (*N* = 26)	
	M1(s)	M2(s)	M1(s)	M2(s)	M1(s)	M2(s)
Psychological flexibility	3.61 (0.92)	3.88 (0.99)	3.66 (1.01)	4.00 (1.00)	3.50 (0.73)	4.04 (0.92)
Time and effort management	2.72 (0.77)	3.29 (0.85)	3.13 (1.03)	3.50 (0.90)	3.25 (0.76)	3.87 (0.70)
Study-related burnout	3.40 (1.19)	3.04 (1.16)	3.16 (1.36)	2.94 (1.09)	3.14 (0.88)	2.62 (0.65)
Exhaustion	3.09 (1.25)	2.69 (1.27)	3.35 (1.38)	3.00 (1.17)	3.25 (1.02)	2.72 (0.80)
Cynicism	3.26 (1.42)	2.80 (1.38)	2.76 (0.155)	2.40 (1.30)	2.54 (1.22)	2.09 (1.00)
Inadequacy	4.27 (0.149)	4.11 (1.26)	3.39 (1.64)	3.61 (1.34)	3.83 (1.17)	3.27 (1.06)

## 5 Discussion

The aim of this study was to explore the students’ change processes during the ACT-based online course using the transtheoretical model as a framework. Our first aim was to identify what kind of change profiles could be identified among the students regarding their perceived benefits of the intervention for their well-being and studying. Four behavior change profiles were recognized representing various stages of the change process. The identified stages closely resembled the transtheoretical model by Prochaska and DiClemente (1982), representing pre-contemplation, contemplation, preparation, and action stages. However, the maintenance stage presented in the original model was not recognized in the present study, since this stage would require sustaining the new behavior over longer term (Prochaska and DiClemente, 1982), and in the present study, the data were collected at the end of the 7-week course. Our results also showed evidence for the importance of psychological flexibility in fostering the change process.

### 5.1 Change profiles in the light of transtheoretical model

Our qualitative analysis showed that there were only few students in the *No changes or insights* profile representing pre-contemplation stage. These students reported not having the resources needed for a change, or they did not recognize the need for a change. Research indicates that individuals in the precontemplation stage tend to focus more on the disadvantages of change rather than its benefits (Prochaska et al., 1994), which could be one reason for not putting effort in the assignments during the intervention. It’s also possible that students in this profile have not considered changing their behavior because they may not perceive their current well-being or study habits as problematic (see also Grunschel and Schopenhauer, 2015).

All the other students reported some changes in thinking or changes in behavior during the course, which is a promising result, and in line with previous studies related to the effectiveness of ACT-based interventions among higher education students (Räsänen et al., 2016; Asikainen and Katajavuori, 2022; Hailikari et al., 2021; Katajavuori et al., 2023; Räihä et al., 2024). One-third of the students in this study reported insights and learning something new during the course. This profile, labelled as *Change in thinking*, aligns with the contemplation stage (Prochaska and DiClemente, 1982). Although these students did not report specific behavioral actions toward improved well-being or study habits, they gained valuable insights into their current behaviors and recognized habits that needed to change or develop. It is likely that the exercises related to thinking of one’s values and goals, being present as well as recognizing one’s thoughts, have supported students in this process. This awareness fostered a shift in their thinking during the intervention. These processes reflect change mechanisms where the need for change is acknowledged, including awareness-raising and self-reflection (Prochaska and DiClemente, 1982; Prochaska et al., 1988; Boonroungrut and Fei, 2018). The students recognized the necessity for change and began to question their behaviors, providing a solid foundation for future action. Ultimately, it has been concluded that individuals often shift their attitudes toward behavior before making actual changes (Prochaska et al., 2008).

Around two thirds of the students reported actual behavior changes during the course. The students represented the *Initial change* profile, representing the preparation stage (Prochaska and DiClemente, 1982). Our qualitative analysis showed that the students in this profile had learnt to understand what needs to be changed and had realized that their own rigid ways of thinking were often the reason for difficulties with their experienced problems. Interestingly, the quantitative analysis revealed that students in the *Initial Change* profile experienced an increase in feelings of inadequacy. In contrast, the other two profiles, which showed changes in behavior or thinking, reported a decrease in inadequacy. This is partly in line with earlier research which has suggested the contemporary phase often comprises negative emotions and possible feelings of worry of failure to change behavior (Prochaska and DiClemente, 1983). These students were already in the precontemplation state but showed small steps towards behavior change. One reason could be that they still were a bit unsure on how to implement changes in their everyday life and felt inadequacy. The shift toward prioritizing the benefits of change over its drawbacks usually occurs during the contemplation or preparation stages (Prochaska et al., 1994). This study suggests that students in the preparation stage may have become more aware of the benefits of changing their behavior. This awareness could help them recognize the need for change more clearly, while also contributing to feelings of inadequacy. The students in this profile likely practiced the sub-processes of defusion and self-as-context, which helped them view their thoughts and behaviors more objectively and question their thinking (Hayes et al., 2006; Hayes et al., 2013). Thus, the difference between this and *Change in thinking* –profile was, that students in this profile had really implemented and practiced changes in their thinking process. Additionally, practicing acceptance skills may have enabled these students to better cope with their negative and frustrating feelings about their ability to change their behavior (Biglan et al., 2008; Blackledge and Barnes-Holmes, 2009). It has been suggested that strengthening self-efficacy and fostering hope in students at the contemplation stage is important (Grunschel and Schopenhauer, 2015). However, our study found that students in the preparation stage would particularly benefit from encouragement.

Around a fourth of the students belonged to the *Change in behavior* profile, reporting significant changes during the intervention. This profile aligns with the action change stage in the transtheoretical model (Prochaska and DiClemente, 1982). Students in this group reported actively working to change their behavior and succeeding in implementing these changes, and it is likely that the value-based action exercises supported this process. Unlike those in the *Initial changes* profile, they did not feel they were just starting out; instead, they focused on the benefits of their changes. This aligns with previous research indicating that individuals in the action stage emphasize the benefits over the drawbacks (Prochaska et al., 1994), suggesting higher confidence in their ability to enact change. This aligns with findings that self-efficacy tends to increase across the stages of change (Grant and Franklin, 2007; Keller et al., 1999). It’s also possible that these students improved their skills in defusion, acceptance, and value-based actions during the intervention, enabling them to focus on the benefits of new behaviors (Hayes et al., 2006; Hayes, 2019). The course included mindfulness practices, which may have enhanced their ability to pay attention to their thoughts. A recent study by Malpass et al. (2019) found that mindfulness training helped students develop a new relationship with their thoughts and feelings, leading to greater control and resilience. By seeing thoughts as just thoughts rather than facts, students were able to distance themselves from negative feelings and respond in healthier ways (Malpass et al., 2019).

The transtheoretical model is valuable because it allows us to identify an individual’s stage of change, enabling timely and appropriate support for facilitating that change (Adams and White, 2005). Understanding university students’ journeys toward improved well-being and studying is crucial for effectively addressing their challenges. Our results indicated that many students gained new insights about themselves, which are essential before actual change can occur. Students identified as belonging to the *Change in thinking* profile should receive encouragement and opportunities to spark their interest in behavior change. Reflecting on these issues can inspire a genuine desire for action. Students in the *Initial change* profile had changes in their thinking but in addition, begun to take small steps toward changing their habits but recognized the significant effort required to improve their study and well-being practices. Therefore, these students would particularly benefit from additional support to help them continue making progress. Different interventions may be needed based on an individual’s current stage of behavior change, and these interventions should align with the processes of change relevant to that stage (Adams and White, 2005). However, the TTM has been criticized for example, for not necessarily capturing behavior change as a continuous process, for assuming a systematic progression through the stages, and for insufficiently accounting for unconscious or external factors that may influence behavior change, or further, to explain how behavior change actually occurs (Sussman et al., 2022). Thus, longitudinal studies are needed to examine the stability of these profiles over time and to further explore these unconscious and external factors that foster behavior change. Important question for future research is whether students classified within the *Change in behavior* profile are able to sustain such extensive behavior changes in the long term and what factors enhance the change from *Change in thinking* to *Initial change* -stage.

### 5.2 Changes in burnout, time and effort management and psychological flexibility across the profiles

The quantitative results revealed that the profiles did not differ from each other at the beginning of the course in terms of psychological flexibility, time and effort management, and study-related burnout. However, in the context of time and effort management and inadequacy, there were bigger differences at the beginning of the course between the profiles than in the other scales, although the differences did not quite reach a statistically significant level. For example, in inadequacy the scores varied between 3.4 (Initial change profile) and 4.3 (Change in Thinking). Thus, the original high score in inadequacy for the *Change in Thinking* profile could have prevented them from making bigger changes in their behavior during the intervention.

When analyzing the effect of time, it was found that two dimensions of burnout, exhaustion, and cynicism decreased during the intervention. Similarly, previous studies have demonstrated that ACT-based interventions can effectively reduce symptoms of burnout (Towey-Swift et al., 2023; Puolakanaho et al., 2020), and through reducing burnout, could also have positive effects on students’ commitment to university studies (Salmela-Aro and Upadyaya, 2017) and academic performance (Asikainen et al., 2022; Madigan and Curran, 2021; Salmela-Aro and Upadyaya, 2017).

The present results further indicated improvements in students’ time and effort management skills. Consequently, the ACT-based intervention proved effective in helping students set goals and study according to those goals, prioritize tasks, and manage their time efficiently (see Katajavuori et al., 2023; Räihä et al., 2024). The positive impact of the ACT-based intervention on these skills may have also contributed to the reduction in students’ study-related burnout, as previous studies have identified a positive correlation between time and effort management and decreased burnout among students (Asikainen et al., 2020; Asikainen and Katajavuori, 2023; Parpala et al., 2021).

Furthermore, the results also suggested that psychological flexibility increased during this course. This improvement may have contributed to the reduced burnout scores, as previous research highlights the importance of psychological flexibility for reducing study-related burnout (e.g., Asikainen and Katajavuori, 2023; Puolakanaho et al., 2020). Furthermore, psychological flexibility is positively associated with academic performance, as well as enhanced time and effort management and self-regulation skills (e.g., Asikainen et al., 2018; Katajavuori et al., 2023; Hailikari et al., 2021; Asikainen and Katajavuori, 2023). Therefore, it is likely that the interplay between time and effort management, psychological flexibility, and positive changes in these areas contributed to the reduction in study-related burnout among students in the present study.

Finally, the questionnaire data suggested that only the changes in inadequacy and psychological flexibility differed significantly between the profiles. In the *Change in thinking* profile, inadequacy decreased but remained higher both before and after the intervention compared to the other two groups. This high feeling of inadequacy may explain why these students did not exhibit behavior changes, likely due to a lack of psychological resources (Schaufeli and Taris, 2014; Salmela-Aro et al., 2022; Bakker et al., 2023). Interestingly, the *Initial change* profile showed an increase in inadequacy, which could be explained by increased awareness of the dimensions of their learning requiring improvement. A similar phenomenon was found among university teachers, who showed a decrease in their self-efficacy beliefs when their awareness of their teaching and areas to be improved increased (see Postareff et al., 2007). The *Change in behavior* profile experienced the biggest decrease in inadequacy; their ability to implement changes in their studying may have fostered positive experiences, thus reducing feelings of inadequacy. Additionally, this profile also showed the largest increase in psychological flexibility, suggesting that these positive changes in psychological flexibility facilitated behavioral improvements, while those behavior changes may have also enhanced their psychological flexibility.

### 5.3 Practical implications for higher education

Overall, our results indicate that the ACT-based course had numerous experienced positive effects on the participating students, although the extent of these changes varied among the students. It has been suggested that universities should strengthen their health promotion policies and activities to meet individual health needs while aligning with the institution’s goal of fostering successful graduates (Baalmann, 2023). This entails providing resources and tools that enhance student well-being, as well as skills that can be learned and practiced. Based on the findings of the present study, ACT-based interventions should be regarded as a valuable approach to enhancing student well-being. This study showed that students progress in their behavior changes differently. Therefore, tailored interventions could be offered according to their stage in the change process. The usefulness of the Transtheoretical Model (TTM) has been demonstrated in earlier studies for tailoring these interventions with assignments that are important and relevant to the individual’s current stage (Armitage and Arden, 2008; Armitage, 2009; DiClemente and Graydon, 2020). The current stage and motivation for a change could be clarified before the intervention, for example with a short survey and reflection assignments. Furthermore, motivation-enhancing goal setting and peer support as used in this intervention can foster the engagement to really make changes in one’s behavior. The integration of practicing the core processes of psychological flexibility should also be incorporated accordingly to one’s current stage. For example, consciousness-raising exercises related to being present, acceptance, and defusion are needed especially in the initial stages of behavior change, whereas exercises related to values, self-as-context, and committed actions are especially essential for changing and maintaining behavior.

For teacher training, the results highlight the value of providing higher education teachers with basic ACT and behavior-change knowledge. Understanding processes such as psychological flexibility or avoidance can help teachers recognize when students struggle with inadequacy or stagnation and respond with supportive pedagogical practices. Integrating ACT-informed approaches into teacher education and continuing professional development could therefore strengthen both teacher well-being and their ability to foster student resilience and self-regulation.

At the policy level, ACT-based approaches could be embedded within broader institutional well-being strategies. For example, universities could integrate short ACT modules into first-year curricula or orientation activities, and design staged support pathways. Because ACT-based exercises are scalable and adaptable, they align well with whole-institution approaches to promoting mental health and academic success. There are examples of ACT-based courses which have been developed for university students and are offered online for all the students in university (Asikainen and Katajavuori, 2022).

### 5.4 Limitations and suggestions for further research

The present study offers valuable insights on higher education students’ change processes during an Acceptance and Commitment Therapy (ACT) based online course. However, the study has its limitations that should be considered when interpreting the results. In terms of the data collection, the quantitative part of the mixed-methods study design comprised of two measuring points, which is a strength. However, the in-depth qualitative analysis was the main aim of this paper, and the quantitative data was only used to complement the qualitative findings. Nevertheless, drawing any causal conclusions on the impact of the intervention based solely on quantitative data cannot be done. Further research with more than two measuring points is needed to study the more the longitudinal changes in students’ behavior, and to be able to research on the *maintenance stage* of the transtheoretical model (Prochaska and DiClemente, 1982), utilized in this study.

Our analysis took into account all the described variations in change; however, it might have been more informative to focus only on concrete actions. On the other hand, while the analysis captured the stages students had reached by the end of the course, it was not able to trace the change process itself during the course. Our data were collected during the 7-week intervention, which does not allow us to make conclusions about the long-term effects of the intervention. In addition, the data was relatively small to analyze the differences of the changes between the profiles. Because of the small effect sizes, the statistical power for these analyses was not strong, and the results should be interpreted with caution. Another thing to consider when interpreting the results is that most likely the students who are at the biggest risk in terms of their study-related well-being do not take part in courses on study skills and well-being. Respectively, students who choose to enroll in such courses are likely to possess pre-existing motivation for self-improvement. This introduces a possible self-selection bias and a potential overrepresentation of well-being–oriented students. However, students represented a heterogenous group based on their background and wellbeing at the beginning of the course. In addition, the purpose of the present study was not to generalize the findings but understand more deeply, how the different level changes and their occurrence in this kind of course. Additionally, it is possible that students’ final reports gave a more optimistic impression on their behavior change than what reality is. Also, it can be estimated that there is a wide variety of ways people write about their individual experiences. The course took place in the Spring of 2022, and thus the influence of COVID-19 pandemic on the results is possible.

In terms of future research, the data of the present study was collected from one country only, and expanding research to include also other cultural contexts would be valuable in the future. Additionally, even if in our study, there were only very few students in a N*o changes or insights* profile representing pre-contemplation stage, it would be beneficial to study more the reasons behind this. In addition, the sample size was small, and the number of students in different change profiles was small and thus, the possibilities to conduct quantitative analyses were restricted. However, the results showed that the profiles and the changes in profiles did not differ from each other. Despite these limitations, the study showed, through a mixed-method design, the positive influence of the intervention on students’ studying and well-being, but also the differences in the changes the individual students reported. Acknowledging the individual differences in how students experience such interventions is important to acknowledge both in future research but also in practical implementation of any efforts to improve student well-being and studying. Future research should also focus on exploring long-term effects of the well-being interventions to understand how maintenance stage is reached and how the changes are maintained. Furthermore, motivational factors as well as social support could be utilized more effectively and their role in fostering change should be explored in future studies. A recent study (Ahmadi et al., 2023) identified practical ways to promote positive change in students’ behavior by supporting their motivation through specific actions, such as providing meaningful choices, clear rationales, opportunities for self-monitoring, empathy, and supportive feedback. This framework (Ahmadi et al., 2023) could be applied in interventions aimed at promoting student well-being and its effects could be explored in forthcoming studies to enhance students’ autonomy, competence, and relatedness, thereby fostering sustained engagement and learning.

## 6 Conclusion

Combining psychological flexibility and transtheoretical frameworks produced interesting and useful insights to explore and foster behavior change among higher education students. This study showed that psychological flexibility fostered behavior change among students and was related to better well-being. The findings provided insights into the diverse change processes among students, highlighting the need for tailored support to encourage behavior change. Future research should examine individual differences to better target interventions for students at different stages of change as well as exploring and fostering long-term effects of this kind of well-being intervention to maintain the desired positive activity.

## Data Availability

The raw data supporting the conclusions of this article will be made available by the authors, without undue reservation.
